# The Alkalinity of the Blood in Gout

**Published:** 1898-06-04

**Authors:** 


### THE ALKALINITY OF THE BLOOD IN GOUT.

It is much to be regretted that a simple question like that of the alkalinity of the blood in gout—a mere problem in chemistry, as one might think—should lead to such heated discussions as are current at the present time. Dr. Haig's opinions on this subject are sufficiently well known. His views are so definite, and his conception of the relation between uric acid on the one hand and gout and its congeners on the other is so neat, and fits in so well with such a number of the clinical facts of those diseases, that many of those who like to have everything scientifically explained not unnaturally lean strongly towards his hypothesis. Still, a question of chemistry must be decided by chemical means. It is not sufficient to deal with alkalies swallowed and with urates excreted; the whole hypothesis hinges upon a varying alkalinity of the blood, and when Dr. Luff asserts, as the result of experiment, that during an attack of gout the alkalinity of the blood does not appreciably differ from that of normal blood, the only effectual answer to such a criticism would seem to be some chemical proof that this observation is wrong. The question is one concerning the chemistry of the blood, not of the urine, and can only be decided by direct analysis of the blood under varying conditions; and the problem is so important, and touches upon the daily work of so many practitioners, that we cannot but feel that it is hardly to the credit of scientific medicine that it should remain an open one any longer. Sarcastic letters in the medical journals are all very well, but the question is one for the chemists.

